# *In Vivo* Dopamine Neuron Imaging-Based Small Molecule Screen Identifies Novel Neuroprotective Compounds and Targets

**DOI:** 10.3389/fphar.2022.837756

**Published:** 2022-03-18

**Authors:** Gha-hyun J. Kim, Han Mo, Harrison Liu, Meri Okorie, Steven Chen, Jiashun Zheng, Hao Li, Michelle Arkin, Bo Huang, Su Guo

**Affiliations:** ^1^ Department of Bioengineering and Therapeutic Sciences and Programs in Biological Sciences and Human Genetics, University of California San Francisco, San Francisco, CA, United States; ^2^ Graduate Program of Pharmaceutical Sciences and Pharmacogenomics, University of California San Francisco, San Francisco, CA, United States; ^3^ Tsinghua-Peking Center for Life Sciences, McGovern Institute for Brain Research, Tsinghua University, Beijing, China; ^4^ Department of Pharmaceutical Chemistry, San Francisco, CA, United States; ^5^ Graduate Program of Bioengineering, San Francisco, CA, United States; ^6^ Small Molecule Discovery Center, University of California San Francisco, San Francisco, CA, United States; ^7^ Department of Biochemistry and Biophysics, University of California San Francisco, San Francisco, CA, United States; ^8^ Chan Zuckerberg Biohub, San Francisco, CA, United States

**Keywords:** neurodegeneration, NTR-MTZ, aloperine, Parkinson’s disease, GBA, gaucher disease, larval screening, zebrafish

## Abstract

Parkinson’s disease (PD) is the second most common neurodegenerative disorder with prominent dopamine (DA) neuron degeneration. PD affects millions of people worldwide, but currently available therapies are limited to temporary relief of symptoms. As an effort to discover disease-modifying therapeutics, we have conducted a screen of 1,403 bioactive small molecule compounds using an *in vivo* whole organism screening assay in transgenic larval zebrafish. The transgenic model expresses the bacterial enzyme nitroreductase (NTR) driven by the tyrosine hydroxylase (th) promotor. NTR converts the commonly used antibiotic pro-drug metronidazole (MTZ) to the toxic nitroso radical form to induce DA neuronal loss. 57 compounds were identified with a brain health score (BHS) that was significantly improved compared to the MTZ treatment alone after FDR adjustment (padj<0.05). Independently, we curated the high throughput screening (HTS) data by annotating each compound with pharmaceutical classification, known mechanism of action, indication, IC50, and target. Using the Reactome database, we performed pathway analysis, which uncovered previously unknown pathways in addition to validating previously known pathways associated with PD. Non-topology-based pathway analysis of the screening data further identified apoptosis, estrogen hormone, dipeptidyl-peptidase 4, and opioid receptor Mu1 to be potentially significant pathways and targets involved in neuroprotection. A total of 12 compounds were examined with a secondary assay that imaged DA neurons before and after compound treatment. The z’-factor of this secondary assay was determined to be 0.58, suggesting it is an excellent assay for screening. Etodolac, nepafenac, aloperine, protionamide, and olmesartan showed significant neuroprotection and was also validated by blinded manual DA neuronal counting. To determine whether these compounds are broadly relevant for neuroprotection, we tested them on a conduritol-b-epoxide (CBE)-induced Gaucher disease (GD) model, in which the activity of *glucocerebrosidase* (GBA), a commonly known genetic risk factor for PD, was inhibited. Aloperine, olmesartan, and nepafenac showed significant protection of DA neurons in this assay. Together, this work, which combines high content whole organism *in vivo* imaging-based screen and bioinformatic pathway analysis of the screening dataset, delineates a previously uncharted approach for identifying hit-to-lead candidates and for implicating previously unknown pathways and targets involved in DA neuron protection.

## Introduction

Neurodegenerative diseases, characterized by progressive loss of neuronal types in the central or peripheral nervous systems (CNS or PNS) followed by multi-organ dysfunction or dementia, are a major source of disability worldwide. Parkinson’s disease (PD) is of particular concern as its prevalence is increasing rapidly but the development of disease-modifying therapeutics has been stagnant (Jankovic and Tan, 2020; Paolini Paoletti et al., 2020). PD is the second most common neurodegenerative disorder that affects more than 10 million people worldwide as of 2020, with an economic burden of $51.9 billion in the United States alone (Yang et al., 2020). Loss of dopamine (DA) neurons in the PD patients results in the cardinal motor symptoms that include bradykinesia, resting tremor, postural instability, and rigidity. Additionally, many PD patients also experience comorbidities including cardiac disorders and increased infection rates that can significantly impede the quality of life and pose severe burdens on their families and caregivers (DeMaagd and Philip, 2015; Armstrong and Okun, 2020). While there are several treatment options for PD that work by enhancing dopamine action, decreasing metabolism of dopamine, or replacing the natural form of dopamine with exogenous drugs tailored for each patient, these therapies provide symptomatic relief only (Armstrong and Okun, 2020). Levodopa is considered the gold standard therapy but is associated with significant complications such as the “wearing off” effect and levodopa-induced dyskinesia. The surgical method with deep brain stimulation has been established for alleviating some of these motor complications and possibly offering neuroprotection in animal models, but the mechanism remains inconclusive (Koprich et al., 2017; Jakobs et al., 2020). Thus, there is an urgent need for identifying disease-modifying therapeutics for PD.

While current therapeutic drug discovery is largely target-based, the implementation of phenotypic drug discovery has significant advantages particularly for neurodegenerative diseases (Ibhazehiebo et al., 2018; Lam and Peterson, 2019; Kim et al., 2021; Zhang et al., 2021). Phenotypic assays for a direct impact on neuronal integrity can bypass the need to fully understand complex biological processes underlying neurodegeneration, and in many cases provide leads to novel targets (Liu et al., 2016; Moffat et al., 2017). By directly imaging brain DA neuronal loss which is the hallmark of PD, our phenotypic screen aims to overcome the current challenge in target-based drug discovery, that is, difficulty in identifying suitable targets for idiopathic conditions. Larval zebrafish is an attractive model for phenotypic drug discovery as it possesses a high degree of genetic, physiological and morphological similarity with humans. Zebrafish genes share 70% homology with human counterparts and 82% disease-related genes have at least one zebrafish orthologue (Howe et al., 2013). The diencephalic region of the zebrafish brain is homologous to the substantia nigra pars compacta in humans which is the region of DA loss in PD patients. DA neurons are readily detectable in larvae as young as 3 days post-fertilization (dpf). zebrafish can produce many embryos on a weekly basis, which can grow up to seven dpf without the need for feeding or handling. The transgenic model used in the screening assay expresses the E. coli nitroreductase (NTR) controlled by the promoter of tyrosine hydroxylase (th), a rate-limiting enzyme in DA synthesis. This model, upon addition of the commonly used antibiotic metronidazole (MTZ), shows robust DA neuronal loss at the larval stage that is suitable for high throughput screening (HTS) screening. Neither genetic models nor neurotoxin (e.g., MPTP) models of PD offer such strength, due to late onset, weak or variable DA neuronal loss. The NTR converts MTZ to the toxic nitroso radical form (Curado et al., 2008; Pisharath and Parsons, 2009; Williams et al., 2015) *in vivo* causing DA neuronal loss that is quantifiable in the ventral forebrain region and involves mitochondrial dysfunction (Kim et al., 2021).

HTS generates large amounts of data and there are many different approaches towards deciding which compounds to pursue further for secondary validation. A widely accepted method for estimating the variability and effect size of the data is through the strictly standardized mean difference (SSMD) (Zhang et al., 2007). While SSMD scores can capture data variability, simply selecting the highest scoring compounds may not be sufficient to uncover candidate hits because SSMD is based on the ratio of mean to standard deviation which could lead to high SSMD scores even with a small mean, resulting in less desirable compounds. Likewise, simply looking at the mean scores (e.g., the brain health scores-BHS) may also result in false positives due to one skewed sample data.

Previously, we developed a high throughput DA neuron imaging method (Liu et al., 2016) and reported the identification of renin-angiotensin-aldosterone system (RAAS) inhibition as neuroprotective via mitochondrial targeting in DA neurons (Kim et al., 2021). In this study, we present for the first time the results of the entire 1403 HTS bioactive compound screen and uncover additional neuroprotective candidates after secondary validation. We apply a multi-pronged approach that incorporates the threshold-based method, topology-based analysis using the Reactome pathway database, and a non-topology-based method. By analyzing the entire screening datasets obtained from the HTS, significant and previously unknown pathways were identified to be involved in neuroprotection.

## Materials and Methods

### Ethics Statement

The study was reviewed and approved by University of California, San Francisco Institutional Animal Care and Use Committee (approval number AN179000). The zebrafish system was regularly inspected by the University of California, San Francisco Laboratory Animal Resource Center.

### Zebrafish Husbandry and Transgenic Lines

For all experiments in the study, homozygous Transgenic *Tg[fuguth:gal4-uas:GFP; uas-NTRmCherry]* and AB wild type were used. Zebrafish were raised on a 14:10 h light/dark cycle and maintained in the zebrafish facility according to the University of California San Francisco Institutional Animal Care and Use Committee standards. Embryos were raised in Blue Egg Water (0.12 g CaSO4, 0.2 g IO Salt, 30 μL of 1% Methylene per L).

### High Throughput Screening of 1,403 Bioactive Compounds

**FIGURE 1:**
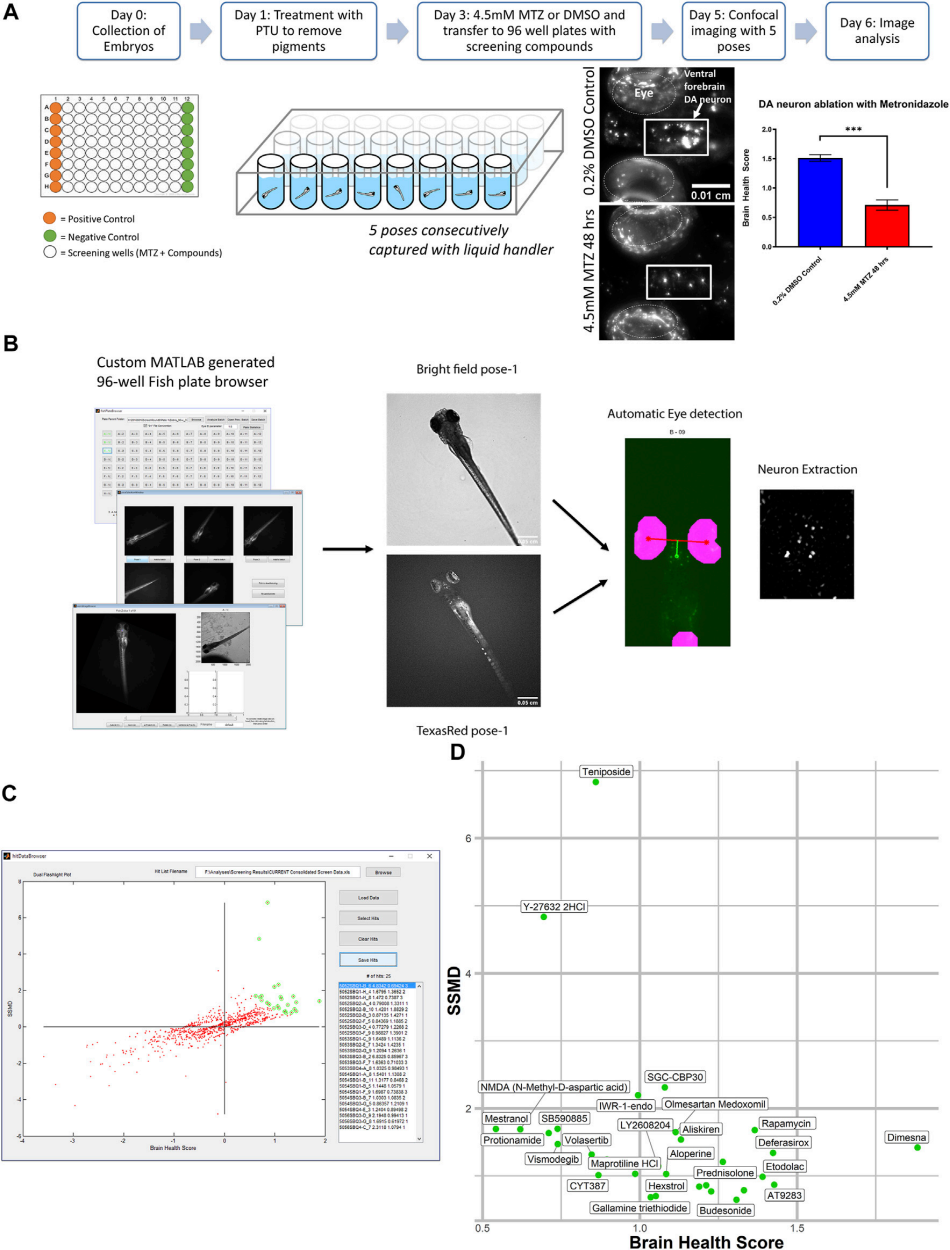
*In vivo* dopamine neuron imaging-based high throughput screening in larval zebrafish identifies potential neuroprotective compounds. **(A)** Overview of the high throughput screening assay. 3 dpf larvae are transferred to 96 well plates with 10 μM screening compounds. DMSO (positive control) or 4.5 mM MTZ (negative control) was added 3 hours later and the treatment lasted for 24 hr, followed by imaging with brightfield and TexRed channels on InCell 6,000. Images were analyzed with the Cellprofiler pipeline. **(B)** Schematic of the image processing pipeline using the custom generated MATLAB “fishplatebrowser” and Cellprofiler. The brightfield and TexRed images were used to automatically detect the eye and diencephalic region of the brain and to quantify DA neurons. **(C)** Dual flashlight plot generated from custom made GUI “HitDataBrowser” with MATLAB. Compounds can be selected and exported with SSMD, BHS, and corresponding sample number. **(D)** Compounds in the top right quadrant with high BHS and SSMD scores based on manual selection. Details of the compounds are shown in Table 1. PTU: 1-phenyl 2-thiourea MTZ: Metronidazole DMSO: dimethyl sulfoxide.

For the *in vivo* high throughput screening assay we utilized a bioactive compound library from SelleckChem obtained from the UCSF Small Molecule Discovery Center (SMDC). As many of these compounds are FDA approved or validated in preclinical research, the target profiles and pharmacodynamics have been established. The assay was performed on a weekly protocol (Figure 1A) spanning from the initial collection of *Tg[fuguth:gal4-uas:GFP; uas:NTRmCherry]* embryos at day 0 and treatment with 200 µM 1-phenyl 2-thiourea (PTU) on 1dpf to remove the pigment. On 3dpf, larvae were transferred to round bottom 96-well plates containing 10 µM of screening compounds and treated with 4.5 mM MTZ for 48 h. The concentration and treatment period of MTZ was determined based on our previous work (Liu et al., 2016), which resulted in robust DA neuron loss (∼60%) without affecting larval zebrafish development and morphology. On 5dpf the larvae were treated with tricaine at a low concentration of 160 ug/mL 30 min prior to imaging the ventral forebrain dopamine (DA) neurons using the InCell 2000 (GE healthcare 28–9,534–63) automated microscope with the TexasRed channel and bright field using a 4 × 0.2NA objective (Nikon) using the built-in 2.5 D deconvolution setting. A total of five different poses were acquired by reorienting the larvae with a liquid handler (Biomek FXp) that mixed 40 μL of the solution in each well to change the orientation.

The images were analyzed on a custom generated MATLAB script (Figure 1B) that allows the manual selection of the best pose and the neurons are automatically extracted using the brightfield images with eyes as landmarks to automatically identify and extract the DA neurons. The analysis was based on a custom CellProfiler (McQuin et al., 2018) pipeline that processes and quantifies the fluorescent intensity and calculates the brain health score (BHS) based on the logarithm of the covariance between the brain image and a reference image generated from multiple healthy brains that was previously described (Liu et al., 2016). The BHS equation is as follows: BHS = log2 Σi,j IijMij, where I is the pixel intensity of the image and M is the pixel intensity of a template image based on the average of 35 brain images at pixel i, j. The SSMD was defined as the ratio of mean to the standard deviation of the difference between the MTZ treated negative control and the sample. The custom pipeline can be found in the Zenodo repository https://doi.org/10.5281/zenodo.5787480. All the experiments were performed in a blinded manner from compound treatment to analysis.

### Topology and Non-Topology-Based Pathway Analysis

**FIGURE 2:**
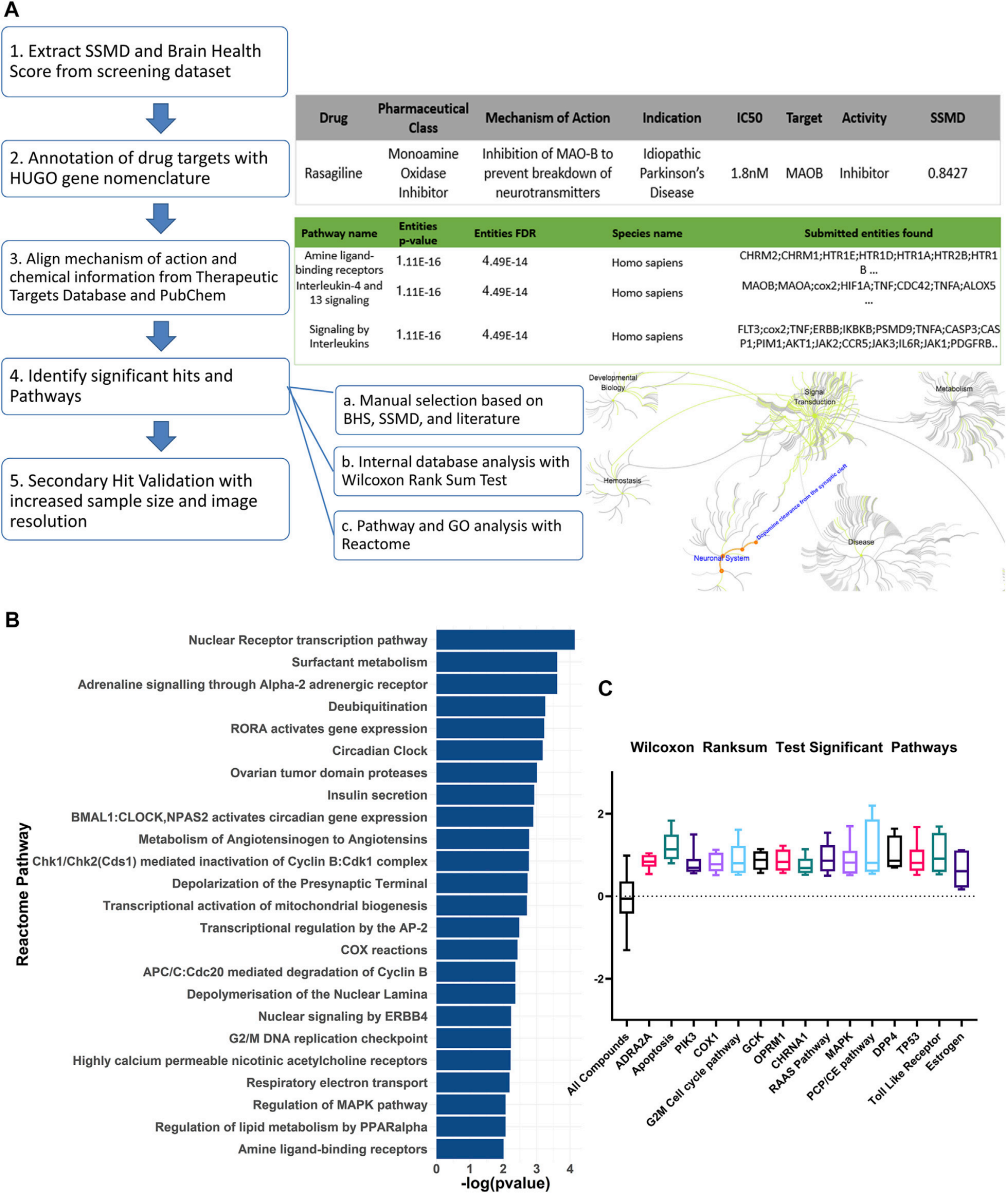
Curation and pathway analysis of the screening dataset identify novel mechanisms of neuroprotection. **(A)** Schematic showing the data processing and analysis pipeline. The example output of the annotations are shown on the right side with the corresponding numbers of each step. Hit calling was based on three criteria, including manual selection with good BHS and SSMD score, Wilcoxon rank sum test, and Reactome pathway analysis. **(B)** A list of significant pathways from the Reactome pathway analysis sorted from highest to lowest significance (Padj <0.01). **(C)** Significant pathways from the non-topology-based pathway analysis of the screening dataset. BHS of the chemicals in the same pathway were compared against BHS of all compounds in the dataset. (*n* = 5 to 13; Padj <0.05, Wilcoxon rank sum test). ADRA2A: Alpha-2A adrenergic receptor, PIK3: Phosphoinositide 3-kinase, COX1: Cytochrome c oxidase subunit I, OPRM1: Mu type opioid receptor, CHRNA1: Cholinergic Receptor Nicotinic Alpha 1 Subunit, RAAS: Renin angiotensin system, MAPK: Mitogen-activated protein kinase, PCP/CE: Planar cell polarity and convergent extension, DPP4: Dipeptidyl peptidase-4, TP53: Tumor protein P53.

The bioactive compound library data was annotated with the Hugo Gene Nomenclature Committee (HGNC) database (Tweedie et al., 2021) and the Therapeutics target database (Wang et al., 2020). For each compound, the pharmaceutical class, known mechanism of action, indication, the half maximal inhibitory concentration (IC50), target, and the activity information was recorded (Figure 2A). For the SSMD and BHS scores of the compounds with opposing mechanisms of action such as inducer versus inhibitor, and agonist versus antagonist, the scores for the compounds with negative SSMD and BHS scores were inverted during the pathway analysis. The Reactome pathway analysis was conducted using the HGNC gene symbols as the identifier and the BHS as the numeric value. The non-topology-based pathway analysis was conducted with the entire HTS dataset. The annotated targets or pathways were analyzed with a Wilcoxon rank sum test to determine whether any had a brain health score that was significantly higher than the average of the entire dataset (FDR adj *p* < 0.05).

### Secondary Assay Optimization and Hit Validation

**FIGURE 3:**
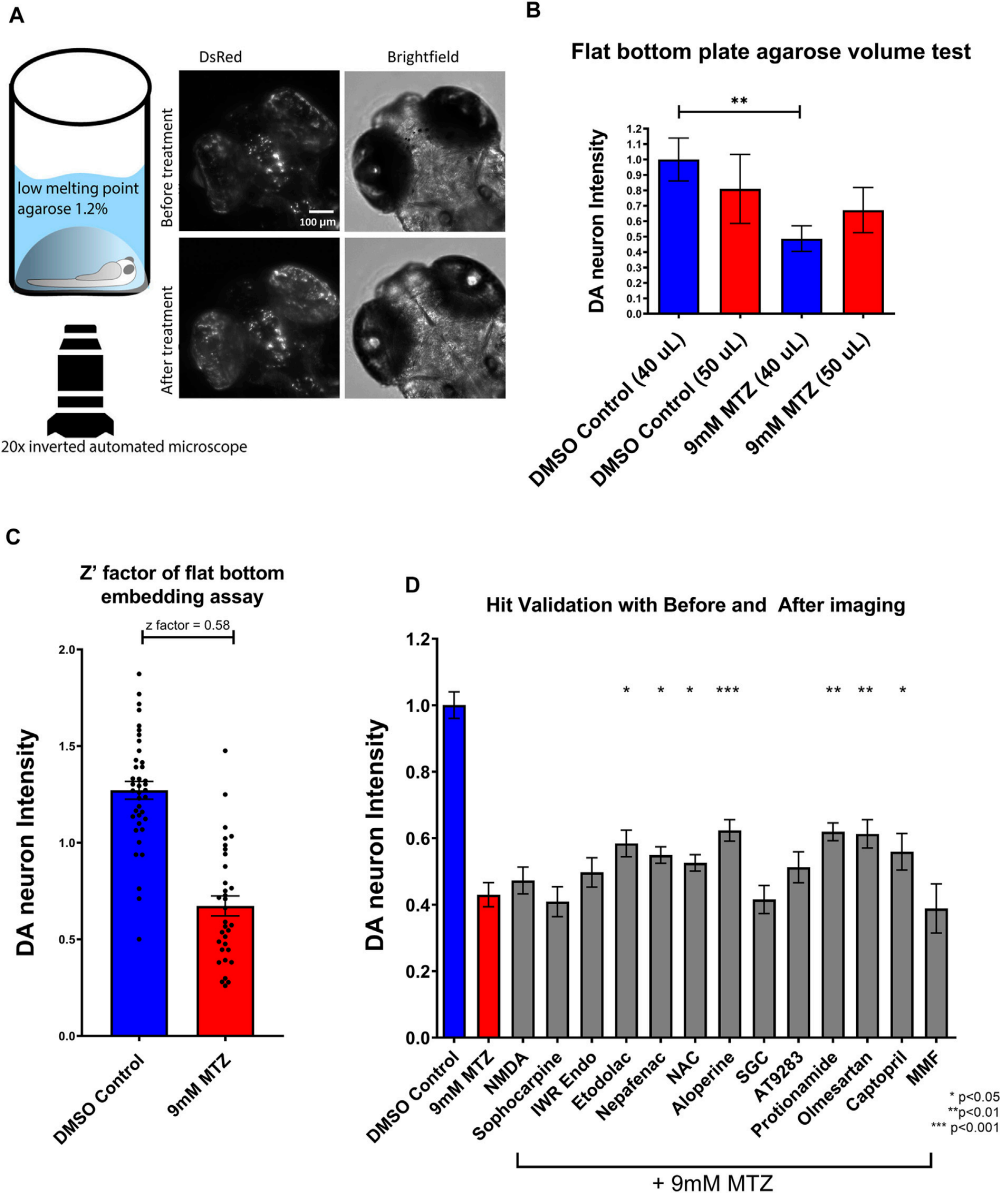
Establishment of a secondary hit validation assay and validation of candidate hit compounds. **(A)** Schematic of the secondary hit validation assay using agarose embedding and automated imaging. At 5 dpf, larvae were embedded in 1.2% agarose and imaged under brightfield and DsRed channels. The larvae were treated with 0.2% DMSO or 9 mM MTZ with or without hit compounds. At 6 dpf, larvae were again imaged with the same x,y,z coordinates on the microscope. Image shown is an example of a 0.2% DMSO control. **(B)** Comparison of 40 and 50 μL 1.2% low melting point agarose for embedding. Samples embedded with 40 μL agarose showed significant difference between DMSO control and 9 mM MTZ (*n* = 8; *p* < 0.05, unpaired *t* test), whereas those with 50 μL agarose did not, due to increased distance between the objective and the samples. **(C)** Evaluation of Z′-factor for the secondary hit validation assay. The 0.2% DMSO control and 24 h of 9 mM MTZ treatment showed a significant difference in DA neuron intensity with a z’factor of 0.58. **(D)** Secondary hit validation of compounds with the embedding assay. Samples were treated with 10 μM of each candidate compound and 9 mM MTZ for 24 h. Etodolac, nepafenac, NAC, aloperine, Protionamide, olmesartan, and captopril showed significantly greater “BHS After treatment” to “BHS before treatment” ratio compared to the negative control (9 mM MTZ) (*n* = 22 to 30; one-way ANOVA F = 12.33, *p* = 0.003, post-hoc Fishers LSD *p < 0.05, **p < 0.01, ***p < 0.001). MTZ: metronidazole, DMSO: dimethyl sulfoxide, NAC: N-Acetyl Cysteine, NMDA: N-methyl-d-aspartate, MMF: Mycophenolate mofetil, SGC: SGC-CBP30.

To validate candidate hit compounds from the primary screen, we developed a medium throughput secondary assay that incorporates larger sample size, higher resolution, and statistical effect size. 5 dpf larvae were embedded in 1.2% agarose and imaged both before chemical treatment and 24 h after treatment, using the same x,y,z coordinates (Figure 3A). Image analysis was conducted by determining the ratio of “after treatment BHS” to “before treatment BHS”. Since embedding did not need the multi-pose method from the initial screen (Liu et al., 2016), a flat bottom 96 well plate was used for greater efficiency in embedding and better tracking of well coordinates and resolution.

The hit candidates selected from the pathway analysis underwent a secondary assay validation with greater sample size. Unlike the primary HTS assay, the secondary assay was designed as a low throughput assay that involves manual embedding of each larval zebrafish in a thin layer of agarose to obtain the most optimal position for visualizing the DA neurons, followed by imaging using a ×20 objective under a confocal microscope with both before and after images taken. The assay was optimized by determining the shape of the well, agarose concentration, and volume of agarose used for embedding. A flat bottom 96 well plate (Griener cat no 655096) was used. The candidate hits were added in 10 μM concentration for 3 h prior to the administration of 9 mM MTZ. The 5 dpf before treatment images were taken on the InCell 6,000 (GE healthcare) and subsequently taken again post 24 h incubation. The images were taken with an inverted ×20 objective under dsRed and brightfield channels (0.45NA, 7.5 mm working distance). 3 μm Z-slices for a total of 40 slices were obtained and the max intensity projection was processed with ImageJ. The BHS was calculated based on the Cellprofiler pipeline as described above. The ratio of BHS before treatment and BHS after treatment was used to quantify the neuroprotective effect and to account for any changes due to brain development during the incubation period. For the dose response studies, concentrations of the compounds were prepared from a series of 5-fold dilutions that were determined by a range based on the known EC50 properties. The candidate compounds were purchased from SelleckChem (NMDA: S7072, Sophocarpine: S2405, IWR Endo: S7086, Etodolac: S1328, Nepafenac: S1255, Aloperine: S2420, SGC-CBP30: S7256, NAC: S1623, AT9283: S1134, Protionamide: S1881, olmesartan: S1604, captopril: S2051, Mycophenolate Mofetil: S1501). The manual screening was performed in a blinded manner by having a single investigator code the compounds and another investigator counting the medium- and large-sized DA neurons under the 20x epifluorescent compound microscope (Zeiss).

## Results

### *In Vivo* DA Neuron Imaging-Based High Throughput Screening Identifies Neuroprotective Compounds

A total of 1,403 bioactive compounds (SelleckChem) were screened at 10 μM concentration that were obtained from the UCSF Small Molecule Discovery Center (SMDC). The dual flashlight plot was created to visualize the strictly standardized mean difference (SSMD) and the BHS (Figures 1C,D). A total of 57 compounds had a BHS score that was significantly greater when compared to MTZ treatment alone (FDR adjusted *p* < 0.05) (Table 1). 67% of the hit compounds identified were inhibitors while 14% were agonists or activators. The remaining compounds were synthetic hormones or glucocorticoids including prednisolone, budesonide, hexestrol, and mestranol. Four compounds were natural products from plants including aloperine, matrine, and sesamin. The primary therapeutic class for the compounds consisted of 32% anti-cancer, 31% neurological, 15% infectious diseases, 12% cardiovascular, and 10% endocrinology drugs.

**TABLE 1:** Top 30 hit compounds from the bioactive high throughput screen with high SSMD and BHS (ranked by BHS).

Compound name	SSMD	Brain health score	*p*-value	Selleckchem ID	Mechanism of action
Dimesna	1.4201	1.8829	0.0120	S1201	Inactivation of acrolein
AT9283	0.8713	1.4271	0.0134	S1134	JAK2/3 kinase inhibitor
Deferasirox	1.3424	1.4235	0.0152	S1712	Iron chelator
Etodolac	0.9883	1.3901	0.0155	S1328	COX inhibitor
Rapamycin	1.6795	1.3652	0.0165	S1039	mTOR inhibitor
AG-490 (Tyrphostin B42)	0.7901	1.3311	0.0167	S1143	EGFR inhibitor
Budesonide	0.6488	1.3074	0.0170	S1286	Glucocorticoid steroid
Prednisolone	1.2094	1.2636	0.0171	S1737	Glucocorticoid steroid
Nepafenac	0.7728	1.2268	0.0176	S1255	COX inhibitor
Sophocarpine	0.8636	1.2109	0.0176	S2405	Tetracyclic quinolizidine alkaloid
Ganetespib (STA-9090)	0.8437	1.1885	0.0203	S1159	HSP90 inhibitor
Aliskiren Hemifumarate	1.5401	1.1308	0.0205	S2199	Direct renin inhibitor
Olmesartan Medoxomil	1.6489	1.1136	0.0208	S1604	Angiotensin II receptor blocker
Aloperine	1.0303	1.0835	0.0210	S2420	PI3K/Akt inhibitor
SGC-CBP30	2.3118	1.0794	0.0222	S7256	CREBBP inhibitor
LY2608204	1.1448	1.0579	0.0238	S2155	Glucokinase activator
Hexstrol	0.7018	1.0509	0.0241	S2473	Nonsteroidal estrogen
Gallamine triethiodide	0.6848	1.0341	0.0336	S2471	Cholinergic receptor blocker
IWR-1-endo	2.1948	0.9941	0.0342	S7086	Wnt inhibitor
Cyproterone Acetate	1.0325	0.9849	0.0365	S2042	Androgen receptor antagonist
Maprotiline HCl	1.2404	0.8950	0.0375	S2517	Noradrenalin reuptake inhibitor
CYT387	1.0129	0.8683	0.0430	S2219	JAK1/2 kinase inhibitor
Teniposide	6.8325	0.8597	0.0403	S1787	DNA topoisomerase II inhibitor
Volasertib (BI 6727)	1.3177	0.8468	0.0411	S2235	Plk1 inhibitor
Vismodegib	1.4720	0.7387	0.0417	S1082	Hedgehog inhibitor
SB590885	1.6987	0.7384	0.0423	S2220	B-raf inhibitor
Protionamide	1.6363	0.7103	0.0432	S1881	Class 1A anti-arrhythmic, Sodium Channel Blocker
Y-27632	4.8342	0.6942	0.0433	S1049	ROCK1 inhibitor
NMDA (N-Methyl-d-aspartic acid)	1.6915	0.6197	0.0436	S7072	NMDA agonist
Mestranol	1.6959	0.5421	0.0447	S2125	Estrogen receptor activation

### Pathway Analyses Identify Previously Unknown and Validate Previously Known Pathways Associated With PD

The Reactome pathway analysis identified 24 significant pathways after correcting for false discoveries (Figure 2B) (*p* < 0.05, FDR = 0.01). With PD being highly related to mitochondrial dysfunction, pathways including deubiquitylation, cyclooxygenase (COX), respiratory electron transport, mitochondrial biogenesis were found to be significant. Other pathways relevant to PD such as acetylcholine receptors, adrenergic signaling, mitogen activated protein kinase (MAPK) were also found to be significant. Additionally, cell cycle and development pathways were found significant including transcriptional regulation by AP-2 and G2/M DNA replication checkpoint. Several pathways were novel or have limited implications in PD, including RAR Related Orphan Receptor A (RORA) gene activation, circadian clock, ovarian tumor proteases, Peroxisome proliferator-activated receptor alpha (PPARα), renin angiotensin system, and insulin regulation.

The non-topology-based pathway analysis using the Wilcoxon rank sum test of the entire dataset showed 15 targets and pathways to be significant (*p* < 0.05, FDR = 0.05) (Figure 1C). Apoptosis, estrogen hormone, dipeptidyl-peptidase 4 (DPP4), and opioid receptor Mu 1 were significant in the Wilcoxon rank sum test but not in the Reactome analysis. A total of 83 compounds were shown to be significant in both the Reactome and Wilcoxon rank sum test (Table 2). 32 compounds were already FDA approved and 20 compounds were in early phase clinical trials.

**TABLE 2:** Significant compounds and pathways identified from the Reactome and Wilcoxon Rank sum test. Detailed information of the 83 compounds from the initial compound library that were shown to be significant in both the Reactome pathway analysis and wilcoxon rank sum test. The strictly standardized mean difference (SSMD) score measures the effect size and the variance amongst the triplicate larval samples for each compound. The brain health score (BHS) was defined as the logarithm of the covariance between the brain image and a template image. During the analysis pipeline, the SSMD and BHS scores were converted for directionality based on the pharmacological activity profile obtained from the Therapeutic Target database. The pathway names were outputted directly based on the target and activity profile from Reactome.

Compound	Pathway name	SSMD	BHS	Target	Activity	FDA status
Dexmedetomidine	Adrenaline signalling through Alpha-2 adrenergic receptor	1.040	−2.928	ADRA2A	AGONIST	Approved
Guanabenz Acetate	Adrenaline signalling through Alpha-2 adrenergic receptor	0.984	−0.868	ADRA2A	AGONIST	Approved
Noradrenaline	Adrenaline signalling through Alpha-2 adrenergic receptor	0.855	−1.021	ADRA2A	STIMULATOR	Approved
Phentolamine Mesylate	Adrenaline signalling through Alpha-2 adrenergic receptor	−0.818	0.624	ADRA2A	INHIBITOR	Approved
Medetomidine	Adrenaline signalling through Alpha-2 adrenergic receptor	0.777	−0.729	ADRA2A	AGONIST	Approved
Ivabradine HCl	Adrenaline signalling through Alpha-2 adrenergic receptor	0.539	0.156	ADRA2A	INHIBITOR	Approved
Y-27632 2HCl	Apoptosis	4.834	0.694	ROCK1	INHIBITOR	
Oprozomib	Apoptosis	1.558	0.221	PSMB8	INHIBITOR	
Apoptosis Activator 2	Apoptosis	1.291	−3.112	CASP3	ACTIVATOR	
Evodiamine	Apoptosis	−1.150	−0.525	BCL2	INDUCER	
RKI-1447	Apoptosis	1.124	−0.097	ROCK1	INHIBITOR	
Dynasore	Apoptosis	0.913	−0.232	DNM1	INHIBITOR	
PF-573228	Apoptosis	0.891	0.305	PTK2	INHIBITOR	
Carfilzomib (PR-171)	Apoptosis	−0.801	−0.066	PSMD9	AGONIST	Approved
ZSTK474	Cell surface interactions at the vascular wall	1.500	0.322	PIK3CA	INHIBITOR	
Dactolisib (BEZ235, NVP-BEZ235)	Cell surface interactions at the vascular wall	0.904	0.571	PIK3CA	INHIBITOR	
RepSox	Cell surface interactions at the vascular wall	0.746	−0.112	TGFB1	INHIBITOR	
Dasatinib	Cell surface interactions at the vascular wall	0.690	0.261	SRC	INHIBITOR	Approved
ML347	Cell surface interactions at the vascular wall	0.625	−0.090	TGFB1	INHIBITOR	
CAL-101 (Idelalisib, GS-1101)	Cell surface interactions at the vascular wall	0.590	0.360	PIK3CA	INHIBITOR	
Bosutinib (SKI-606)	Cell surface interactions at the vascular wall	0.558	0.134	SRC	INHIBITOR	Approved
Ibuprofen (Dolgesic)	COX reactions	1.124	0.217	COX	INHIBITOR	Approved
Mefenamic acid	COX reactions	1.074	0.446	COX	INHIBITOR	Approved
Etodolac (Lodine)	COX reactions	0.988	1.390	COX	INHIBITOR	Approved
Bromfenac	COX reactions	0.778	1.053	COX	INHIBITOR	Approved
Nepafenac	COX reactions	0.773	1.227	COX	INHIBITOR	Approved
Diclofenac Sodium	COX reactions	0.694	0.428	PTSG2	INHIBITOR	Approved
Ketorolac (ketorolac tromethamine)	COX reactions	0.577	0.504	COX	INHIBITOR	Approved
Suprofen (Profenal)	COX reactions	0.510	0.423	COX	INHIBITOR	Approved
Enzastaurin (LY317615)	Depolymerisation of the Nuclear Lamina	0.522	0.610	PRKCB	INHIBITOR	
JTC-801	G-protein activation	−1.223	−3.519	OPRM1	ANTAGONIST	
Matrine ((+)-Matrine)	G-protein activation	0.800	0.787	OPRM1	AGONIST	
Naloxone HCl	G-protein activation	0.564	0.964	OPRM1	AGONIST	Approved
Tenovin-1	G2/M DNA damage checkpoint	−1.612	−0.715	TP53	ACTIVATOR	
VE-821	G2/M DNA damage checkpoint	0.923	0.332	ATM	INHIBITOR	
VE-822	G2/M DNA damage checkpoint	0.781	0.041	ATR	ANTAGONIST	
LY2608204	Glycolysis	1.145	1.058	GCK	INHIBITOR	
Clorsulon	Glycolysis	0.907	0.518	GPM1	INHIBITOR	
Vismodegib (GDC-0449)	Hh mutants that don’t undergo autocatalytic processing are degraded by ERAD	1.472	0.739	SHH	INHIBITOR	Approved
PNU-120596	Highly calcium permeable nicotinic acetylcholine receptors	−1.142	−3.868	CHRNA1	AGONIST	
Tropicamide	Highly calcium permeable nicotinic acetylcholine receptors	0.952	0.697	CHRNA1	INHIBITOR	Approved
Darifenacin	Highly calcium permeable nicotinic acetylcholine receptors	0.869	0.064	CHRNA1	INHIBITOR	Approved
Pancuronium dibromide	Highly calcium permeable nicotinic acetylcholine receptors	0.860	0.930	CHRNA1	INHIBITOR	Approved
Gallamine triethiodide (Flaxedil)	Highly calcium permeable nicotinic acetylcholine receptors	0.685	1.034	CHRNA1	INHIBITOR	Approved
Adiphenine	Highly calcium permeable nicotinic acetylcholine receptors	0.671	0.306	CHRNA1	INHIBITOR	
Bethanechol chloride	Highly calcium permeable nicotinic acetylcholine receptors	0.570	−0.201	CHRNA1	AGONIST	Approved
Atropine sulfate monohydrate	Highly calcium permeable nicotinic acetylcholine receptors	0.551	0.416	CHRNA1	INHIBITOR	Approved
Cytisine	Highly calcium permeable nicotinic acetylcholine receptors	−0.520	−0.750	CHRNA4	AGONIST	
Aliskiren hemifumarate	Metabolism of Angiotensinogen to Angiotensins	1.540	1.1308	REN	INHIBITOR	Approved
Imidapril HCl	Metabolism of Angiotensinogen to Angiotensins	0.938	0.5801	ACE	INHIBITOR	
Enalapril Maleate	Metabolism of Angiotensinogen to Angiotensins	0.860	2.947	ACE	INHIBITOR	
Quinapril hydrochloride (accupril)	Metabolism of Angiotensinogen to Angiotensins	0.707	0.385	ACE	INHIBITOR	Approved
Ramipril	Metabolism of Angiotensinogen to Angiotensins	0.498	0.253	ACE	INHIBITOR	Approved
SB590885	Negative feedback regulation of MAPK pathway	1.699	0.738	RAF1	INHIBITOR	
Selumetinib (AZD6244)	Negative feedback regulation of MAPK pathway	1.098	0.172	MEK1	INHIBITOR	
RAF265 (CHIR-265)	Negative feedback regulation of MAPK pathway	0.886	0.537	RAF1	INHIBITOR	
SL327	Negative feedback regulation of MAPK pathway	0.812	0.668	MEK1	INHIBITOR	
Vemurafenib (PLX4032, RG7204)	Negative feedback regulation of MAPK pathway	0.625	0.962	BRAF	INHIBITOR	Approved
Tanshinone IIA (Tanshinone B)	Negative feedback regulation of MAPK pathway	0.547	1.823	MAP2K1	INHIBITOR	
PD0325901 (PD325901)	Negative feedback regulation of MAPK pathway	0.511	0.597	MEK1	INHIBITOR	
IWR-1 (endo-IWR 1)	PCP/CE pathway	2.195	0.994	WNT1	INHIBITOR	
EHop-016	PCP/CE pathway	0.879	−0.273	RAC1	INHIBITOR	
XAV-939	PCP/CE pathway	0.544	0.853	WNT1	INHIBITOR	
Protionamide	Peptide hormone metabolism	1.636	0.710	INHA	INHIBITOR	
Alogliptin	Peptide hormone metabolism	0.988	0.720	DPP4	INHIBITOR	Approved
TAK-875	Peptide hormone metabolism	0.733	1.320	gpr40	ANTAGONIST	
SGC-CBP30	Regulation of Hypoxia-inducible Factor (HIF) by oxygen	2.312	1.079	DOT1L	INHIBITOR	
Rapamycin	Regulation of TP53 Activity	1.679	1.365	MTOR	INHIBITOR	Approved
P22077	Regulation of TP53 Activity	1.145	0.694	USP7	INHIBITOR	
ETP-46464	Regulation of TP53 Activity	1.085	0.023	MTOR	INHIBITOR	
Ridaforolimus	Regulation of TP53 Activity	1.078	0.298	MTOR	INHIBITOR	
PP242	Regulation of TP53 Activity	0.896	0.892	MTOR	INHIBITOR	
KU-0063794	Regulation of TP53 Activity	0.618	1.254	MTOR	INHIBITOR	
PHT-427	Regulation of TP53 Activity	0.616	0.553	AKT1	INHIBITOR	
Entinostat (MS-275)	Regulation of TP53 Activity	0.574	0.524	HDAC1	INHIBITOR	
AZD1152-HQPA (Barasertib)	Regulation of TP53 Activity	0.517	1.01	AURKB	INHIBITOR	
Carprofen	Respiratory electron transport	0.697	0.858	cox2	INHIBITOR	Approved
Cilengitide	Smooth Muscle Contraction	0.718	−0.104	ITGA1	INHIBITOR	
(-)-Huperzine A	Synthesis of PC	1.320	0.550	ACHE	INHIBITOR	
Odanacatib (MK 0822)	Toll-Like Receptors Cascades	−1.054	−0.098	CTSK	AGONIST	
EUK 134	Toll-Like Receptors Cascades	0.529	0.279	APP	INHIBITOR	
NMDA	TP53 Regulates Metabolic Genes	1.691	0.619	NMDA	AGONIST	
BAM 7	TP53 Regulates Transcription of Genes Involved in G2 Cell Cycle Arrest	0.763	0.027	BAX	INDUCER	

### Secondary Hit Validation Identifies Non-Steroidal Anti-inflammatory Drugs, Renin Angiotensin System, Aloperine, and Protionamide to Be Neuroprotective

For secondary hit validation, we developed an agarose embedding method to achieve better resolution for imaging DA neurons. We first experimented with the volume of agarose used for embedding. 40 μL was chosen as it did not harm or stress larvae during the 24 h incubation period (Figure 3B). The higher 50 uL volume of agarose resulted in worsening image quality due to the distance between the DA neurons and inverted objective lens. Furthermore, having too much agarose possibly resulted in less air exchange, thus impairing the health of the larvae when embedded for a prolonged period of time. The calculated z’ factor of the secondary assay was 0.58, which is considered an excellent assay with less within-group variation compared to the z’ factor 0.35 of the primary assay (Figure 3C, Supplementary Figure S1).

**FIGURE 4:**
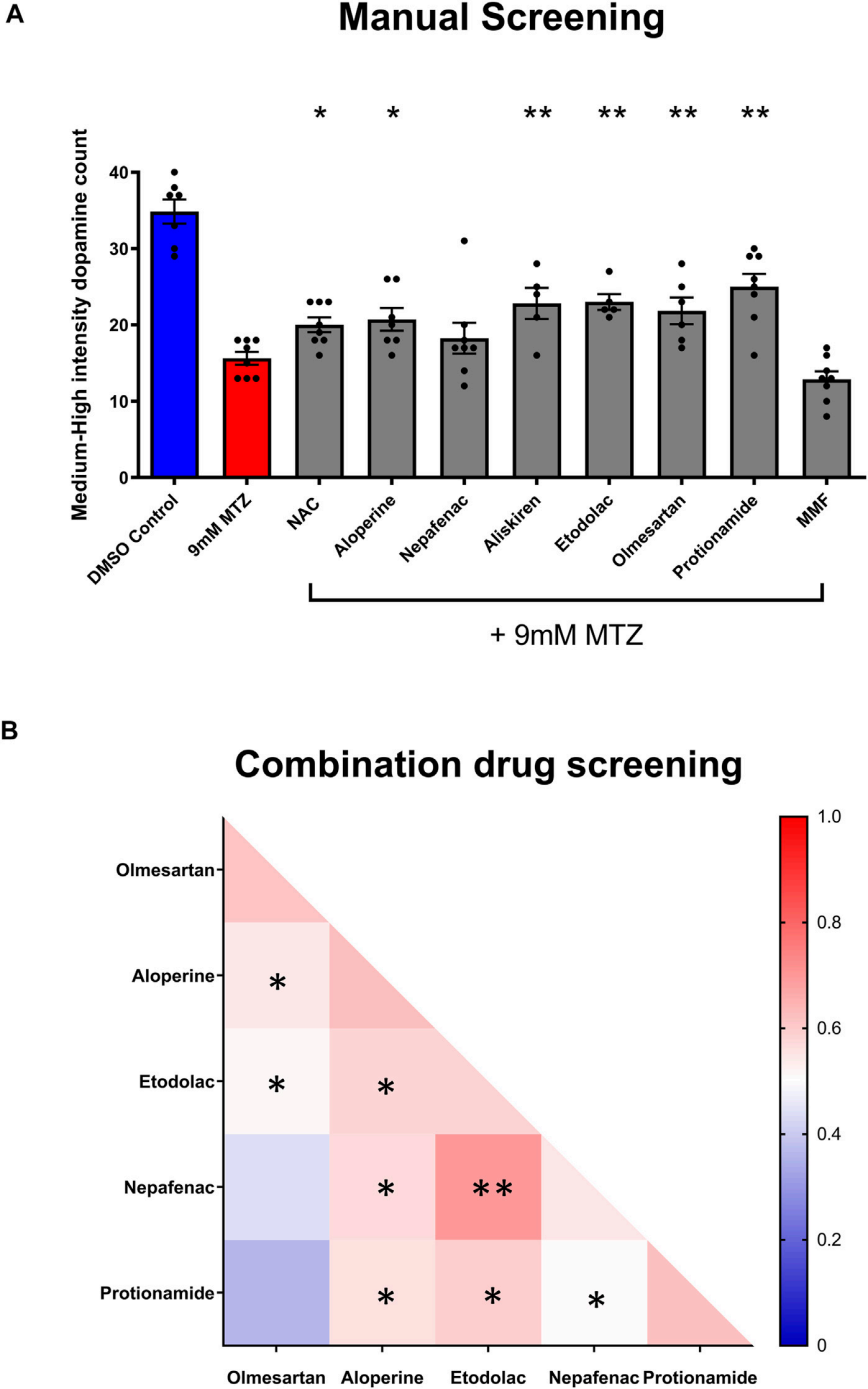
Manual screening and combination screening of hit candidates based on secondary assay. **(A)** Manual screening of the significant compounds identified from the secondary hit validation assay. All samples were manually quantified in a blinded manner after 24 h treatment with candidate compounds and MTZ as described above. (*n* = 7 to 8; one-way ANOVA F = 16.72, *p* < 0.001, post-hoc Fishers LSD *p < 0.05, **p < 0.01). **(B)** Heatmap matrix showing the BHS for testing hit compounds in combination. All candidate compounds were 10 μM in concentration. The combination of etodolac-nepafenac, etodolac-protionamide, and etodolac-aloperine showed greater BHS compared to the administration of either alone. 0.2% DMSO for positive control and 9 mM MTZ for negative control. (*n* = 12 to 16; *p < 0.05, **p < 0.01, unpaired *t* test).

Utilizing the secondary hit validation assay, a total of 12 candidate compounds were tested for neuroprotection. We selected these compounds based on a combination of statistical thresholding using SSMD and BHS and pathway analyses. Additionally, N-Acetyl Cysteine (NAC) was used as a reference compound based on previous studies showing significant neuroprotection in other DA models (Monti et al., 2019). After treatment with 9 mM MTZ for 48 h and comparing the BHS of the before and after images, 10 μM etodolac, nepafenac, aloperine, NAC, protionamide, olmesartan, and captopril showed significant neuroprotection (Figure 3D). These compounds were then manually validated in a single blinded design by counting the medium to high intensity dopamine neurons after 24 h of MTZ treatment. All compounds except for nepafenac were shown to be significant compared to control (*p* < 0.05) (Figure 4A). A dose response study of nepafenac showed lower doses (0.04 and 2.0 μM) to be neuroprotective (Supplementary Figure S2). For the dose response study, there were no linear dose response relationships observed in the BHS scores and toxicity was observed for all compounds at the highest concentrations.

Significantly neuroprotective drugs were also tested in combination to determine the possible drug pairs that could provide additive or synergistic effects on neuroprotection. The combination of etodolac-nepafenac, etodolac-protionamide, and etodolac-aloperine showed a greater BHS compared to the administration of either compound alone (Figure 4B).

### Validation of Candidate Compounds in a Chemically Induced Gaucher Disease Model Uncovers DA Neuron Protection

**FIGURE 5:**
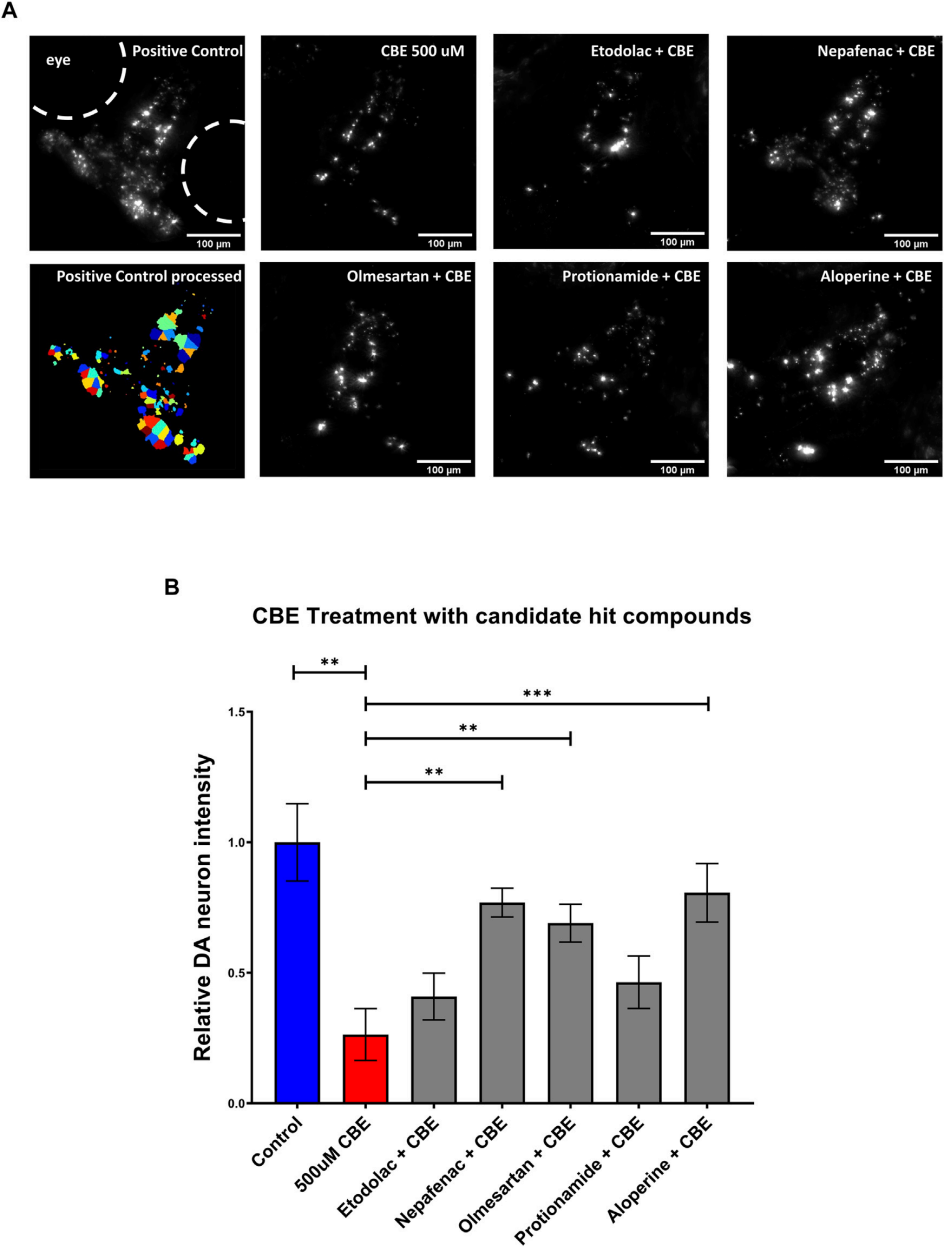
Validation of candidate compounds in a chemically induced Gaucher disease model. **(A)** High throughput imaging of DA neurons with the InCell 6,000 platform for the positive control, CBE, and the candidate compounds. The bottom left image shows the DA neuron isolation process in the custom Cellprofiler pipeline used for image analysis. **(B)** Hit validation of candidate compounds with 48 h treatment of 500 μM CBE. At 5 dpf, larvae were treated with 0.2% DMSO (positive control), 500 μM CBE (negative control), and the CBE+ 10 μM candidate compounds for 48 h. At 7 dpf, the larvae were imaged with a confocal microscope. The 500 μM CBE showed significant reduction in DA neurons compared to the 0.2% DMSO control (N = 12; *p* = 0.0012, unpaired *t*-test). Nepafenac, olmesartan, and aloperine showed significant neuroprotection when co-treated with CBE (*N* = 10 to 12; one-way ANOVA F = 6.205, *p* < 0.001, post-hoc Fishers LSD **p < 0.01, ***p < 0.001, unpaired *t*-test). CBE: Conduritol B epoxide.

As the NTR-MTZ induced DA neuron degeneration does not directly relate to the etiology of PD in humans, we next tested the candidate compounds using a chemically induced Gaucher’s disease model. Gaucher’s disease involves mutations in the glucocerebrosidase (gba1) gene, which is known to be the most common genetic risk factor for PD (Riboldi and Di Fonzo, 2019). Chemical inhibition of GBA was achieved using conduritol B-epoxide, which has been previously established in both mice and zebrafish (Vardi et al., 2016; Artola et al., 2019). 5 dpf larvae were treated with 10 μM of the candidate compounds etodolac, nepafenac, olmesartan, protionamide, and aloperine along with 500 μM CBE for 48 h. At 7 dpf, the larvae were imaged with the InCell 6,000 high throughput confocal imaging platform and the ventral DA neurons were analyzed with the custom CellProfiler pipeline (Figure 5A). The compounds nepafenac, olmesartan, and aloperine showed significant neuroprotection compared to the CBE treatment alone (*p* < 0.01, *p* < 0.01, and *p* < 0.001 respectively, one-way ANOVA with post-hoc Fischer’s LSD) (Figure 5B).

### Statistical Analysis

The SSMD and BHS data from high throughput screening studies were analyzed by one-way ANOVA and post-hoc Fishers LSD (lease squares difference) test with the R program and is expressed as means ± SEM unless otherwise stated. When only two groups were present (i.e., DMSO versus MTZ control or 40uL agarose versus 50uL agarose), an unpaired *t*-test was performed. The pathway analysis with Reactome was conducted with an over-representation (hypergeometric) test. The non-topology-based pathway analysis was carried out using the Wilcoxon rank sum test to identify significant targets from the entire screen. All the secondary hit validations were conducted with a one-way ANOVA and post-hoc LSD between the sample and negative control (MTZ treatment).

## Discussion

By developing a whole organism screening assay that directly images DA neurons of larval zebrafish in a high throughput manner, we have introduced a phenotype-based method for identifying compounds that protect against DA neuron degeneration. The secondary hit validation assay that utilizes the embedding technique to image before and after treatment showed an excellent z’ factor score. Since a threshold-based hit calling method using SSMD and BHS scores focuses primarily on the selection of top scored hits, this is limited due to the small sample size of *n* = 3 in the primary screen, some true hit may be missed due to low affinity of the compounds that may be improvable by future medicinal chemistry. We therefore employed additional bioinformatic analysis to select candidates based on significant results from the pathway analysis. These efforts led to the identification of new pathways previously not linked to PD, as well as the validation of pathways previously implicated in PD.

Pathway analysis revealed mitochondrial dysfunction and respiratory transport chain pathways that are known to be closely tied to the etiology of PD (Park et al., 2018). This finding further strengthens the relevance of our screening assay to PD. The relevance of our screening assay has also been established in our recent report of an in-depth analysis of the RAAS inhibitors in PD (Kim et al., 2021). Etodolac and nepafenac identified in our screen are known COX-2 selective inhibitors, which have been previously studied as potential PD therapeutics with its anti-inflammatory properties. Particularly COX-2 is involved in microglia activation, production of radicals, and protects against DA neuron loss in 6-OHDA rat models (Sánchez-Pernaute et al., 2004; Bartels and Leenders, 2010).

Pathways that are not previously associated with PD could lead to new targets and therapeutics. The pathway related to circadian rhythm regulation was found significant from the Reactome pathway analysis. These include the BMAL1:CLOCK:NPAS2 circadian gene expression pathway. Sleep disturbance is a common nonmotor complaint in PD but the etiology is not well understood (Breen et al., 2014). The circadian clock gene BMAL1 is important in sleep control and leukocytes of PD patients have shown to have altered expression that also correlates with PD severity (Cai et al., 2010). Studies in mice have shown that cholinergic neurons of the basal forebrain are more active in Bmal1 muscle-overexpressed mice (Ehlen et al., 2017). In zebrafish, circadian genes modulate dopamine levels (Huang et al., 2015). Insulin regulation and glucose control was also found to be significantly linked to neuroprotection in the pathway analysis. This is supported by a previous report that hyperglycemia increases the production of reactive oxygen species from the mitochondrial electron transport chain and type 2 diabetes is associated with an increased risk of PD (Hu et al., 2007).

The natural product aloperine showed strong and validated neuroprotective effects in this study. Aloperine is a quinolizidine-type alkaloid that is known to have antioxidant properties through suppression of NF-κB signaling (Xu et al., 2014), activation of nuclear factor erythroid-related factor 2 (Song et al., 2018). Aloperine can also inhibit apoptosis in amyloid induced mouse cells in a mitochondria-dependent pathway (Zhao et al., 2018). The neuroprotective benefits of natural compounds are a promising topic of interest but further efforts on elucidating their pharmacokinetic and pharmacodynamic properties are needed (Sharifi-Rad et al., 2020).

The initial screen had a relatively low sample size of *n* = 3 which could have led to variability and potential false errors. However, this was mitigated by calculating the SSMD score and evaluating not single compounds, but a group of compounds based on pharmacological targets and pathway analysis. Furthermore, secondary validation was conducted with greater sample size along with a blinded manual screen. With the NTR/MTZ assay, it is possible that the compounds that act as inhibitors of NTR could come across as being neuroprotective. These compounds should not show neuroprotection in the second model, the CBE-induced GD model. Therefore, by using both assays in our study, we shall be able to identify broad neuroprotective compounds and distinguish them from NTR inhibitors. The hit compounds identified in this paper will require follow up studies in other animal models and at the mechanistic levels to understand their potential neuroprotective effects in PD.

## Data Availability

The original contributions presented in the study are included in the article/Supplementary Material, further inquiries can be directed to the corresponding authors.
